# Editorial: The early life window of opportunity: role of the microbiome on immune system imprinting

**DOI:** 10.3389/fimmu.2024.1417060

**Published:** 2024-05-03

**Authors:** Amir Ardeshir, Thomas Gensollen, Melody Zeng, Ziad Al Nabhani, Richard Blumberg

**Affiliations:** ^1^ Tulane National Primate Research Center, School of Medicine, Tulane University, Covington, LA, United States; ^2^ Department of Microbiology and Immunology, School of Medicine, Tulane University, New Orleans, LA, United States; ^3^ Institut de Pharmacologie et de Biologie Structurale (IPBS), Université de Toulouse, CNRS, Université Toulouse III - Paul Sabatier (UT3), Toulouse, France; ^4^ Department of Pediatrics, Gale and Ira Drukier Institute for Children’s Health, Weill Cornell Medicine, Cornell University, New York, NY, United States; ^5^ Department of Visceral Surgery and Medicine, Bern University Hospital, University of Bern, Bern, Switzerland; ^6^ Maurice Müller Laboratories, Department for Biomedical Research, University of Bern, Bern, Switzerland; ^7^ Division of Gastroenterology, Department of Medicine, Brigham and Women’s Hospital, Harvard Medical School, Boston, MA, United States

**Keywords:** early-life microbiome, immune system development, window of opportunity, health span, immunological imprinting, microbiota-targeted interventions, probiotics, dysbiosis

The early stages of life, from the late fetal period to infancy, represent a critical window of opportunity for shaping the immune system and setting the stage for lifelong health span. During this time, the developing microbiome plays a crucial role in educating and priming immune responses, with long-lasting consequences for health and disease. This Research Topic brings together five articles that provide novel insights into the complex interactions between the early-life microbiome and immune system development. **Figure 1** provides a visual representation of the key concepts discussed in this Research Topic, highlighting the critical role of the early-life microbiome in shaping health span.

**Figure 1:**
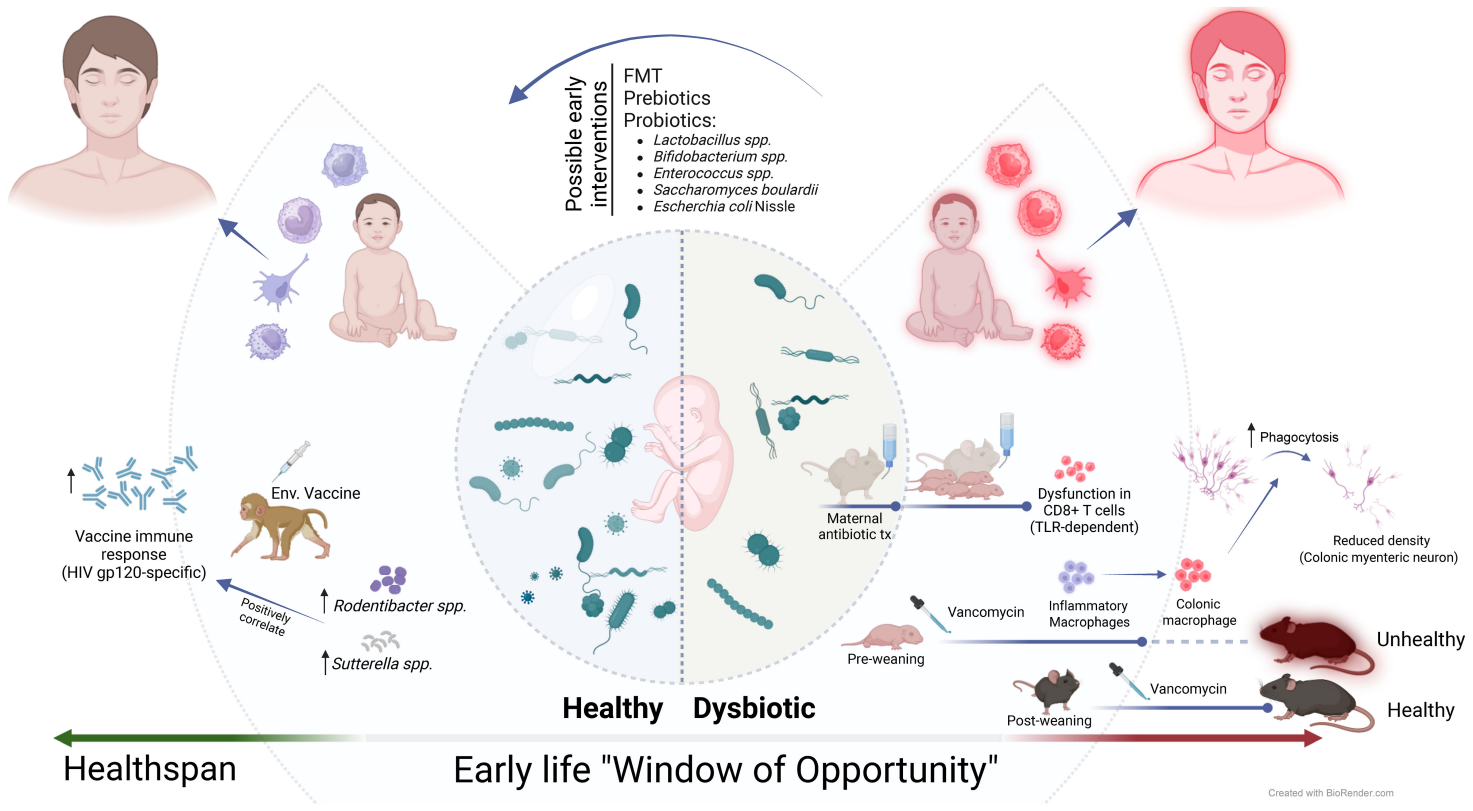
The early-life microbiome plays a crucial role in shaping health span. A healthy microbiome, characterized by diverse beneficial bacteria, promotes optimal immune function and gut-brain communication. However, early perturbations caused by antibiotic treatment can lead to dysbiosis, resulting in reduced enteric neuron density, increased inflammatory macrophages, and dysfunction in CD8+ T cell function in mouse models. Interestingly, a more robust vaccine-elicited immune response correlates with the prevalence of some genera in rhesus macaques. Maintaining a balanced early-life microbiome is essential for promoting lifelong health and preventing the development of an “unhealthy span”.

To set the stage for understanding the impact of the early-life microbiome on immune function, Reynolds and Bettini‘s review delineates the pivotal role of neonatal microbiota in orchestrating immune system ontogeny, emphasizing the modulation of immune maturation and tolerance by early microbial encounters. It elucidates the influence of perinatal factors—antibiotic administration, mode of delivery, and nutritional inputs—on microbiome composition and the consequent predisposition to immunopathologies. The review critically evaluates microbiota-centric interventions, including fecal microbiota transplantation, prebiotics, and probiotics, for rectifying early-life dysbiosis. Highlighting the necessity for advanced research, it advocates for precision in microbial restoration techniques to mitigate the onset of allergies and autoimmune disorders, thereby underscoring the microbiome’s integral contribution to immunological homeostasis during neonatal development. Building upon this foundation, Gonzalez-Perez and Lamousé-Smith demonstrate the far-reaching effects of early-life microbial perturbations on immune function. They discovered that maternal antibiotic treatment (MAT) during late pregnancy and lactation significantly alters the gastrointestinal microbiota composition in infant mice, leading to altered CD8+ T cell function. MAT infant CD8+ T cells exhibit diminished interferon gamma (IFN-γ) production, altered expression and activation of key T cell receptor (TCR) signaling proteins, and compromised effector function. Remarkably, lipopolysaccharide treatment partially restored IFN-γ production in MAT effector CD8+ T cells and reduced mortality following viral infection. These findings underscore the importance of maintaining a healthy microbiome during early life to ensure optimal T cell function and immune responsiveness, which may impact health span.

Delving deeper into the specific mechanisms by which the microbiome influences immune development, Yang et al. explore the effect of the microbiome on intestinal innate immune development in early life. They discuss how the microbiome influences the maturation of innate immune cells, such as macrophages and dendritic cells, and the development of barrier function. The authors also propose potential early intervention strategies, including probiotics and prebiotics, to promote a healthy microbiome-immune interaction. These insights provide a framework for future research aimed at developing targeted interventions to support optimal immune development and health span.

Moving beyond the gut, Schill et al. reveal how neonatal exposure to the antibiotic vancomycin disrupts gut microbiota, leading to significant changes in the gut-brain-immune axis during critical postnatal development in mice. Specifically, vancomycin treatment results in an increased presence of pro-inflammatory colonic macrophages by promoting the recruitment of bone-marrow-derived macrophages, and a concurrent reduction in enteric neuron density within the myenteric plexus. Remarkably, these effects are confined to the neonatal phase, highlighting a vulnerable developmental window where the gut microbiome plays a pivotal role in shaping the enteric nervous system (ENS) and immune responses. The study underscores the necessity of reconsidering antibiotic usage in early life, due to its potential long-lasting impacts on gut health and beyond. This study highlights the broader implications of antibiotic use in early life and the need to consider the gut-brain-immune axis when evaluating the consequences of microbiome perturbations.

Finally, Jiang et al. explore the translational potential of employing the early-life microbiome to enhance vaccine efficacy. They identify *Sutterella* and *Rodentibacter*, two genera of bacteria, as positively correlating with antibody responses to vaccines in infant rhesus macaques. Specifically, *Sutterella* and its associated metabolic pathways are positively associated with HIV gp120-specific IgG responses induced by an HIV envelope (Env) vaccine. This finding highlights the potential of leveraging specific microbial taxa and their metabolic functions to enhance vaccine efficacy in early life, opening new avenues for targeted microbiome-based interventions.

Collectively, the articles in this Research Topic contribute to our growing understanding of the intricate relationship between the early-life microbiome and immune system development. The three original research articles provide valuable insights into specific microbial taxa, metabolic pathways, and immune cell interactions that shape immune responses and have long-term consequences for health. The review articles offer a comprehensive overview of the current state of knowledge in this field, highlighting the importance of maintaining a balanced microbial community for optimal immune function and health span. The findings presented in this Research Topic underscore the need for further research to fully elucidate the complex interplay between the microbiome, immune system, and other physiological systems, such as the enteric nervous system. Future studies should focus on identifying targeted microbiome-based interventions, such as the use of pre- and probiotics or the judicious use of antimicrobials, to promote a healthy microbiome-immune interaction during the critical early-life period.

In conclusion, this Research Topic emphasizes the significant role of the early-life microbiome in shaping immune system development and its potential impact on lifelong health outcomes. The articles within this Research Topic contribute to the growing body of evidence supporting the importance of the early-life window of opportunity for microbiome-immune interventions. As we continue to unravel the complexities of the early-life microbiome-immune interaction, collaborative efforts between researchers and clinicians will be crucial in translating these findings into practical strategies for promoting optimal immune development and disease prevention.

## Author contributions

AA: Conceptualization, Project administration, Resources, Supervision, Visualization, Writing – original draft, Writing – review & editing. TG: Conceptualization, Supervision, Writing – review & editing, Project administration. MZ: Project administration, Supervision, Writing – review & editing. ZN: Project administration, Supervision, Writing – review & editing. RB: Project administration, Supervision, Writing – review & editing.

